# Supplementary material for: Collaborative intelligence in AI: Evaluating the performance of a council of AIs on the USMLE
Source: PLOS Digit Health. 2025 Oct 9;4(10):e0000787. doi: 10.1371/journal.pdig.0000787 (PMC12510544; doi:10.1371/journal.pdig.0000787)
Supplement: S2 File — (ZIP) [file pdig.0000787.s002.zip › S2 Data Raw data files containing unprocessed question inputs and LLM outputs including transcripts of deliberations/STEP3_Question-71-1_Type-MC-NJ.docx]

**Facilitator** **[Round 1]:** A 40-year-old man comes to the office for a preemployment physical examination. The patient has been generally healthy. Medical history is unremarkable and he takes no routine medications. Vital signs are normal. Physical examination shows a palpable nodule in the right lobe of the thyroid gland. Serum thyroid-stimulating hormone concentration is within the reference range. Ultrasonography of the thyroid gland confirms a solid, 1-cm nodule. Which of the following is the most appropriate next step in evaluation? (A) CT scan of the neck (B) Fine-needle aspiration of the nodule (C) Radionuclide thyroid scan (D) Thyroidectomy (E) Observation only

**AI_E:** RESTATING THE QUESTION: The query here is about the next appropriate diagnostic step for a 40-year-old man who was discovered to have a palpable, solid 1-cm nodule in the right lobe of his thyroid gland during a routine pre-employment physical. The nodule was confirmed via ultrasonography. His thyroid-stimulating hormone (TSH) levels are normal, and he has no significant medical history or routine medication use. PLANNING: The first assumption is that the nodule might be malignant. However, another assumption could be that the nodule is benign. Differentiating between these assumptions is crucial since it determines the next steps in patient management. In clinical scenarios, the preferred method of evaluating thyroid nodules is typically dependent on the patient's thyroid function status and the sonographic characteristics of the detected nodule. CLINICAL REASONING: In a case where the patient's serum TSH levels fall within the reference range (referred to as "euthyroid"), the use of fine-needle aspiration (FNA) is recommended as the first step for the evaluation of solitary thyroid nodules, regardless of their size. FNA is a minimally invasive procedure that can provide valuable cytological information to distinguish between benign and malignant nodules. The decision for FNA is also based on the sonographic characteristics of the nodule, and a solid nodule, regardless of its size, is a candidate for this procedure. If the FNA result is benign, the patient can be reassured and observed with periodic follow-up. Conversely, if the FNA result is malignant or suspicious, the patient undergoes thyroidectomy. CONCLUDING: Considering the patient's clinical and sonographic findings as well as euthyroid status, it is reasonable in this situation to prioritize the assumption that the nodule might be malignant. Therefore, the evaluation should progress to cytologic analysis via fine-needle aspiration. RESPONSE: Hence, the most appropriate next step in this patient's evaluation is (B) Fine-needle aspiration of the nodule.

**AI_B:** (1) RESTATING THE QUESTION: The situation here involves a 40-year-old man who is presenting for a routine physical examination as a part of his pre-employment check-up. He's been generally healthy, with no remarkable medical history or medications. His physical examination has revealed a palpable nodule in his right thyroid lobe. His thyroid-stimulating hormone (TSH) levels are normal, and an ultrasonography has confirmed the presence of a 1 cm solid nodule in his thyroid. The question is asking us to identify the most appropriate next step in his medical evaluation from a range of options including a CT neck scan, a fine-needle aspiration, a radionuclide scan, a thyroidectomy, or merely observation. (2) PLANNING: Starting with the assumptions, the fact that the patient presents a palpable nodule in the thyroid gland means that the situation could possibly include benign or malignant conditions. The nodule being solid and 1 cm in size further implies the need for evaluation. The normal TSH levels mean that the nodule is nonfunctional, providing additional information. Thus, the presence of a solid, palpable thyroid nodule necessitates further evaluation to rule out malignancy. Among the options provided, B, fine-needle aspiration of the nodule, stands out because it is a cost-effective, minimally invasive procedure that can determine if the nodule is benign or malignant. (3) CLINICAL REASONING: Clinical guidelines indicate that for thyroid nodule of size >1cm with normal TSH, a fine-needle aspiration biopsy is the recommended next step. The reasoning behind this is primarily to identify any signs of malignancy. A palpable thyroid nodule carries a 5-15% risk of malignancy and therefore needs evaluation. A fine-needle aspiration provides us with cytological examination and has high sensitivity and accuracy for malignancy detection. Other options such as CT scan, radionuclide thyroid scan or thyroidectomy are not typically used as first-line investigation in such scenarios, and an observation-only approach would not be appropriate given the potential risk of malignancy. (4) CONCLUDING: Therefore, following the clinical guidelines and considering the effectiveness of the assessments and potential risk factors, I conclude that the fine-needle aspiration of the nodule 'option B' would be the most appropriate next step in evaluating the thyroid nodule detected in this otherwise healthy individual. (5) RESPONSE: The most appropriate next step in evaluation for this patient is (B) Fine-needle aspiration of the nodule.

**AI_D:** (1) RESTATING THE QUESTION: The case involves a 40-year-old male who has been found to have a solid, palpable nodule in the right thyroid lobe. Despite the presence of the nodule, his thyroid-stimulating hormone (TSH) levels are normal, which suggests that the nodule is non-functioning. The question now is regarding the best subsequent step to evaluate this thyroid nodule. (2) PLANNING: While all of the options listed are potential assessments for thyroid nodules, the best next step in the clinical evaluation should be determined based on the current available evidence. Here, the assumptions can be that the nodule is either malignant or benign. A malignant nodule requires more aggressive investigation, while a benign nodule might only require monitoring. As the precise nature of the nodule is unknown, the most conservative assumption, i.e., considering the nodule as potentially malignant, should be prioritized to avoid missing a cancer diagnosis. (3) CLINICAL REASONING: Now, to evaluate our assumption of the thyroid nodule being potentially malignant, it's necessary to use an approach that can directly assess the nodule's cytology. In this context, CT scans and radionuclide scans can provide information about the size, location, and metabolic activity of the nodule, but not about its cytology, which is crucial for differentiating between benign and malignant cells. Observation alone would not yield any more data and would delay potential treatment if the nodule were indeed malignant. Thyroidectomy would definitively determine the nature of the nodule but is too invasive a procedure to be done without a prior biopsy. On the other hand, fine-needle aspiration (FNA) is a procedure that allows for cytological examination of the cells within the nodule and can therefore differentiate between a cancerous and a benign nodule. (4) CONCLUDING: Therefore, in the clinical evaluation of a thyroid nodule, especially with normal TSH levels like in the current situation, fine-needle aspiration would be the most appropriate step as it allows for direct cytologic examination, making it possible to differentiate between benign and malignant nodules. Furthermore, it's a less invasive procedure compared to a thyroidectomy and provides more actionable information than what a CT scan, radionuclide thyroid scan, or observation alone could. (5) RESPONSE: Thus, the most appropriate next step in evaluation of the 1-cm solid thyroid nodule in this 40-year-old man would be option (B) Fine-needle aspiration of the nodule.

**AI_C:** RESTATING THE QUESTION: The question presents a 40-year-old man with a solid palpable thyroid nodule that is detected during a routine physical examination. The patient is otherwise healthy. Thyroid-stimulating hormone (TSH) levels are within normal limits. A thyroid nodule is usually a growth within the thyroid gland. TSH is a hormone secreted by the pituitary gland that stimulates the thyroid gland to produce thyroid hormones. The question asks for the most suitable next step in evaluating this patient. PLANNING: The primary considerations here are deciding whether there's need for immediate surgical intervention, observation only, a more detailed imaging study, or biopsy of the concerning nodule. The presence of a nodule does not necessarily indicate malignancy, and many thyroid nodules are benign. Hence, making a decision to proceed straight to thyroidectomy, i.e., surgical removal of the thyroid, seems an excessive measure. An acronym to remember in this kind of scenario is 'SIRAD' which stands for Size, Involvement (infra/extra thyroidal), Risk factors, Age, and Doppler characteristics with S, I, and D being the ultrasound findings that prompt a fine-needle aspiration biopsy. CLINICAL REASONING: Imaging like CT scan or radionuclide scan doesn’t usually add any additional beneficial information in such cases where ultrasonography already confirms the presence of a solid nodule. The role for a radionuclide scan would be in cases with hyperthyroidism which is not applicable to this case. Observation might not be the best course since a solid and palpable nodule in the thyroid could indicate a malignancy. Although thyroid nodules are common and usually benign, potential malignancy should be evaluated, particularly in the case of a solid nodule. CONCLUDING: Therefore, the most appropriate next step would be to confirm the nature of the nodule. This would generally involve a fine-needle aspiration (FNA) of the nodule for cytological evaluation in order to determine whether it's benign or malignant. This procedure is typically performed under ultrasound guidance and provides a cellular sample of the thyroid nodule. It is considered to be the most reliable method for evaluating thyroid nodules, having a high sensitivity and specificity for diagnosing malignancy. RESPONSE: So the best response would be (B) Fine-needle aspiration of the nodule.

**AI_A:** 1) RESTATING THE QUESTION: The question is essentially about what would be the next course of action in diagnosing a healthy 40-year-old man who, upon physical examination, presents with a palpable nodule in the right lobe of his thyroid gland. The concentration of his thyroid-stimulating hormone (a hormone responsible for stimulating the thyroid to produce thyroxine), tests within the normal range and an ultrasonography has confirmed the presence of this solid, 1-cm nodule. 2) PLANNING: In addressing this question, we may consider multiple options: CT scan of the neck, fine-needle aspiration of the nodule, radionuclide thyroid scan, thyroidectomy, or observation only. A CT scan would provide detailed images of the neck structures, including the thyroid gland. Fine-needle aspiration, on the other hand, would help to collect cells from the nodule for cytological examination. A radionuclide thyroid scan would showcase the function of the thyroid gland specifically at the nodule region, while a thyroidectomy would be the process of removing the entire thyroid gland. Finally, observation would imply a "wait and watch" approach. 3) CLINICAL REASONING: In the analysis of solitary thyroid nodules, the primary concern is the possibility of the nodule being a thyroid cancer. The most reliable method for establishing the diagnosis is fine-needle aspiration (option B). It will allow us to examine the cells within the nodule closely and establish a firm diagnosis. Option C (radionuclide scan) is not a typical first step unless a patient exhibits a suppressed TSH (typically seen in hyperthyroidism) which is not the clinical picture here. Options A (CT scan of neck) and D (Thyroidectomy), though potentially useful, are typically more applicable after an initial suspicious or unclear fine needle aspiration result. 4) CONCLUDING: Therefore, considering the thyroid gland's normal function and the presence of an isolated solid nodule, a fine-needle aspiration would be the most suitable first step in evaluation. It is more effective, less invasive, and cost-efficient than most other fits. 5) RESPONSE: The most appropriate next step in evaluating this patient would be fine-needle aspiration of the nodule (option B). This procedure will help establish whether the nodule is benign or malignant and guide future management plans.

**Facilitator** The consensus among the AI responses is that the most appropriate next step in evaluating a 40-year-old man with a palpable, solid 1-cm nodule in the right lobe of his thyroid gland, confirmed by ultrasonography, is to perform a fine-needle aspiration of the nodule (option B). This decision is based on the fact that the patient's thyroid-stimulating hormone (TSH) levels are within the normal range, indicating that the nodule is non-functioning. The primary concern with solitary thyroid nodules is the potential for malignancy. Fine-needle aspiration is a minimally invasive procedure that allows for cytological examination of the cells within the nodule, providing the most reliable method for differentiating between benign and malignant nodules. It is considered the first-line investigation in such scenarios due to its high sensitivity and accuracy for malignancy detection. Other options such as a CT scan of the neck, radionuclide thyroid scan, or thyroidectomy are typically not used as the initial step in evaluation. A CT scan or radionuclide scan would not provide additional beneficial information in this case where ultrasonography has already confirmed the presence of a solid nodule. A thyroidectomy, while definitive, is too invasive a procedure to be done without a prior biopsy. An observation-only approach would not be appropriate given the potential risk of malignancy. Therefore, considering the clinical and sonographic findings, the patient's euthyroid status, and the need to rule out malignancy, the consensus is that the most appropriate next step in this patient's evaluation is a fine-needle aspiration of the nodule.
